# Community Use and Perceptions of Climate Shelters in Schoolyards in Barcelona

**DOI:** 10.3389/ijph.2025.1608083

**Published:** 2025-02-07

**Authors:** Marta Sanz-Mas, Xavier Continente, Marc Marí-Dell’Olmo, María José López

**Affiliations:** ^1^ Servei d’Avaluació i Mètodes d’Intervenció, Agència de Salut Pública de Barcelona, Barcelona, Spain; ^2^ Departament de Ciències Experimentals i de la Salut (DCEXS), Universitat Pompeu Fabra, Barcelona, Spain; ^3^ Grupo de Evaluación de políticas y programas de salud pública, Consorcio de Investigación Biomédica en Red de Epidemiologia y Salud Pública (CIBERESP), Madrid, Spain; ^4^ Grupo de Evaluación de políticas y programas de salud pública, Institut de Recerca Sant Pau (IR SANT PAU), Barcelona, Spain

**Keywords:** climate change adaptation, community, schools, thermal comfort, urban health

## Abstract

**Objectives:**

This study explores the use and perceptions of thermal comfort and wellbeing among the local community in the schoolyards in Barcelona that had been transformed into accessible climate shelters.

**Methods:**

We conducted a qualitative evaluation through a spontaneous ethnographic approach, combining 22 non-participant observations in the 11 transformed schoolyards with brief spontaneous interviews with 76 users and 18 caretakers who supervised the schoolyards during non-school days (June–July 2022). We conducted a thematic content analysis. We analyzed user characteristics, thermal comfort and wellbeing, activities and differences by age and gender, social behavior, additional benefits, and areas for improvement.

**Results:**

Adapted schoolyards were mostly visited by families and groups of adolescents with a higher proportion of boys. Participants reported that shade provided coolness and comfort, vegetation offered pleasant and naturalized environments, and water features were enjoyed for their cooling capacity and play opportunities. Schoolyards were mainly used as places for outdoor recreation and socialization.

**Conclusion:**

This study provides policymakers with evidence to support the transformation of schoolyards into climate shelters, creating comfortable outdoor recreational areas for the surrounding urban community.

## Introduction

Climate change has exacerbated the urban heat island effect in the Mediterranean region, leading to hotter summers and more frequent heatwaves [1] This phenomenon negatively affects human health, increasing the risk of hospitalization [2] and heat-related mortality [3]. Urban areas face particular threats due to the lack of green spaces [4]. These challenges highlight the urgent need for effective nature-based mitigation and adaptation strategies to create healthier and more resilient cities.

As part of its Climate Plan, Barcelona City Council has created a network of climate shelter spaces within the city, adapting urban public spaces to provide residents with thermal comfort. These areas are designed to be easily accessible, safe, and provide comfortable rest areas and free water [5]. As part of this strategy, 11 public primary schoolyards in Barcelona were transformed into climate shelters through the “Climate Shelters in Schools” project. This involved implementing vegetation (trees, green walls, and planters with Mediterranean species), water features (drinking fountains, fountains for playing and cooling, and evaporative misters), shade structures (pergolas and canopies), and seating areas (benches and tiered seating). Each schoolyard was adapted according to its characteristics, potential, and needs [6, 7]. These areas were also integrated into the “School Playgrounds Open to the Neighborhood” program, which aims to make the schoolyards in Barcelona available to the public outside school hours (weekends, holidays, and summer).

Transforming schoolyards into climate shelters is a promising strategy for enhancing urban climate resilience [8–10]. Implementing vegetation, natural surfaces, and shade structures in schoolyards can provide cooler environments, reducing outdoor thermal discomfort and heat stress [11–14]. Green spaces also have the ability to improve mental wellbeing, quality of life, and general health [15–17]. Additionally, incorporating water infrastructure offers potential for drinking and cooling [11].

Climate-adapted schoolyards are not just potential spaces for shelter but can serve as accessible community areas that promote physical activity and outdoor play in natural settings [18] and provide opportunities for social interaction, fostering social cohesion [18, 19]. In addition, schools are strategically located throughout the city, making them easily accessible to large numbers of people, ensuring that the benefits of the intervention are equally distributed throughout the city.

Schoolyard transformations have already proven beneficial for the school community by improving thermal comfort during school hours and promoting play diversification and social inclusion during recess [20]. The present study aims to understand the potential benefits of this intervention beyond the school community, exploring the use and perceptions of thermal comfort and wellbeing of the transformed schoolyards as accessible climate shelters among the local community in Barcelona.

## Methods

### Study Design

We conducted a qualitative evaluation study using a spontaneous ethnographic approach, combining non-participant observations and brief spontaneous interviews. The transformations were carried out between July and August 2020. After the implementation, we collected data during non-school periods (summer June–July 2022) as part of the “School Playgrounds Open to the Neighborhood” program.

### Study Setting

The study area comprised the 11 schoolyards in Barcelona that had been transformed as part of the “Climate Shelters in Schools” project and were opened to the community. These schools had been selected previously based on their higher climate change vulnerability while ensuring representation of all city districts [7].

### Measures

We used a spontaneous ethnographic approach, consisting of non-participant observations combined with brief semi-structured interviews. Non-participant observation involves observing participants and their behavior without actively interacting with them. Photographs with some examples of the interventions implemented in the schoolyards were captured during the observation sessions (Supplementary Figure S1).

We performed a total of 22 observations in the 11 schoolyards that underwent the intervention (2 sessions/school). Observations lasted approximately 1.5 h and were conducted on 2 days of the week (1 weekday and 1 weekend day) and in the afternoon during the non-school period (June–July 2022). The schoolyard was divided into various target areas, each defined by the type of equipment available. Target areas were defined before the observations and the same target areas were observed in both sessions. Data were collected through a field diary and included information on the number of users and their characteristics, such as age, gender, and type of relationship between group members (family, friendship, alone). We defined 6 age groups: younger children (aged 0–5 years), older children (aged 6–11 years), adolescents (aged 12–18 years), young adults (aged 19–35 years), middle-aged adults (aged 36–55 years), and older adults (aged >55 years). We also analyzed users’ activities and types of play, social behavior, and use of the schoolyard equipment, materials, and areas (paying special attention to the use of shaded areas, interaction with greenery, and use of water facilities). We also gathered data on temperature, relative humidity, and weather (sunny/cloudy/rainy) using a weather app. Shade coverage and the perception of thermal comfort according to the observers were also collected in the various target areas.

We conducted and audio-recorded 3- to 5-minute interviews with 76 users who were spontaneously selected during observations. We aimed to include diversity by selecting different profiles, particularly regarding age and gender. Interviews were conducted individually or in groups. We also interviewed 18 caretakers who supervised the schools during opening hours. Initially, we gathered information about respondents’ age and gender. We adopted the same age group classification as that used in the observational data. We then assessed their previous experience with the schoolyard prior to the intervention (yes/no). Users were also asked whether they lived in the neighborhood (yes/no), belonged to the school community (yes/no), and were aware of the role of the schoolyard as a climate shelter (yes/no). The interviews conducted with schoolyard users included questions on how often they visited the schoolyard and their main reasons for doing so. Caretakers were interviewed about users’ main characteristics, activities, and reasons for using the schoolyard, as well as their perception of thermal comfort and wellbeing at the schoolyard and differences in schoolyard use according to users’ age and gender. Both users and caretakers were asked to provide their opinions on shaded areas, vegetation, and water features, report the features they most appreciated about the schoolyard, and propose suggestions for improvement. Respondents were also asked to rate the suitability of the schoolyard as a climate shelter on a 1 to 10 scale.

### Data Analysis

Interviews were transcribed and complemented with the interviewer’s field notes. Observation notes and interview transcripts were analyzed following a thematic content analysis. Assisted by ATLAS.ti software, we coded the data thematically and subsequently grouped the emerging codes into different categories and sub-categories. We adopted a grounded theory approach, allowing codes and categories naturally arise from the data and refining our coding frame throughout the data analysis process. Three members of the research team with biomedical, medical, and pharmaceutical background were involved in the coding process. Codes and categories were agreed upon by all members of the research team. We analyzed each technique separately and converged the data during the interpretation of the results.

For the observations, we described the observed activities, social behavior, the presence of shade, and the observer’s thermal comfort. We analyzed these factors for different user characteristics and for each type of schoolyard area, including resting areas (i.e., places designed for relaxation and peacefulness, which include shade structures, seats, or both, and may also have areas with vegetation), sports courts, playground areas (i.e., sandpit, climbing wall, or slides), and water features. We also calculated the median temperature and median relative humidity of the observation days.

For the interview transcripts, six categories emerged from the thematic analyses: 1) users’ characteristics; 2) thermal comfort and wellbeing; 3) activities and differences by age and gender; 4) social behavior; 5) additional benefits; and 6) areas for improvement. We also calculated the caretakers’ and users’ mean scores on their perception of the suitability of the schoolyard as a climate shelter.

## Results

### Non-Participant Observations

Temperatures during the observation sessions ranged from 27°C to 30°C (median = 29°C), relative humidity ranged from 17% to 52% (median = 45%) and the weather was mostly sunny. We observed a total of 263 community members (90 groups) using the climate shelters, with the number of users ranging from 0 to 24 depending on the school and day. In most of the schoolyards, we observed a higher number of men and boys (n = 168) than women and girls (n = 90). The gender of 5 individuals was not recorded. We also observed individuals from different age groups, mainly younger children (n = 33), older children (n = 67), adolescents (n = 57), and middle-aged adults (n = 86). Occasionally, we observed young adults (n = 9) and older adults (n = 11).

Table 1 shows the results from the observational data regarding the use of the schoolyard and thermal characteristics by schoolyard area. Areas designed for resting (i.e., areas with shade structures, seats, which may or may not include vegetation) and children’s areas (i.e., sandpit, climbing wall, slides, etc) had total or partial shade, while the sports court areas had partial or poor shade coverage. Shade was provided mainly by shade structures, trees, and the school building. Observers’ perceptions of thermal comfort were more favorable in the areas with more shade, such as the resting and children’s areas compared to the sports court.

**TABLE 1:** Observational data on schoolyard use and thermal characteristics. Presence of shade, observer’s thermal comfort, user characteristics, activities and social behavior by schoolyard area. Climate Shelter in Schools, Barcelona, 2018–2022.

Schoolyard area	Shade coverage and observer’s thermal comfort	Age group	Activities by gender	Social behavior
Resting areas (including shade structures and/or seats)	Total or partial shade coverage (trees, green wall, shade structures, building) • Average thermal comfort score: 7.5	Children (younger and older)	• Girls and boys: sitting, eating, jumping • Mainly girls: skating	• Solitary or social play • Peer interaction
		Adolescents and young adults	• Girls and boys: talking, sitting, using mobile phones, listening to music	• Verbal interaction with other users • Peer interaction
		Middle-aged and older adults	• Women and men: sitting, resting, talking, reading, writing, using electronic devices, watching their children, viewing others’ activities	• Verbal interaction with other users
Sports court	Partial or poor shade coverage (building, trees, shade structures) • Average thermal comfort score: 4.6	Children (younger and older)	• Girls and boys: ball sport games (younger children), running, frisbee • Mainly boys: ball sport games (older children), riding a scooter • Mainly girls: skating	• Social play • Parent-child interaction • Interaction with children of similar and different ages
		Adolescents and young adults	• Mainly boys: ball games, riding a scooter	• Social play • Peer interaction
		Middle-aged adults	• Mainly men: playing with their children	• Parent-child interaction
Playground areas (i.e., sandpit, slide, climbing wall)	Total or partial shade coverage (building, shade structures, trees) • Average thermal comfort score: 8.4	Children (mainly younger)	• Girls and boys: sandpit, slide, frisbee, climbing, interacting with nature, chasing games • Mainly girls: balancing, building games, bowling, pétanque	• Solitary or social play • Peer interaction
		Middle-aged adults	• Women and men: watching their children, sitting, resting, talking to other adults, playing with their children	• Verbal interaction with other users • Parent-child interaction
Water features	Not applicable	Children (younger and older)	• Girls and boys: Playing and cooling with water, drinking water	• Solitary or social play • Peer interaction
		Adolescents and young adults	• Mainly boys: drinking water, cooling down with water	• No interaction
		Middle-aged adults	• Women and men: little use of the fountains	• No interaction

Resting areas were popular among middle-aged adults, who mainly sat in the shade, chatting with other adults, and watching their children. Children, adolescents, and young adults used the shaded and seating areas to rest and sit briefly between active play periods. Children also used these areas as places to eat or engage in vigorous activities such as jumping or skating, either alone or with their peers. Adolescents and young adults used these spaces for quieter activities including chatting with friends, using mobile phones, or listening to music.

Sports courts were one of the most frequently used areas. Children, adolescents, and young adults played sports games with balls, especially football and basketball. Younger children, regardless of gender, were observed in the court, while older children, adolescents, and young adults who were playing in this area were mostly boys. Children also engaged in other activities in this area, such as running, playing frisbee, and skating (mainly girls). We observed social play between same-age groups as well as mixed-age groups.

Children’s areas were mostly used by younger children and their parents. Children played alone or with their peers in the sandpit, on slides, at the climbing wall, or occasionally interacted with nature. Parents watched their children or talked to other adults, while sitting under the shade.

Fountains were mainly used by children, adolescents, and young adults. Children used them to play, cool off, and drink water. Adolescents and young adults, mostly boys, used them to drink water and cool off during short breaks from their main activity, usually football.

### Brief Spontaneous Interviews

#### User Characteristics

Respondents’ characteristics are shown in Table 2. Most caretakers (72.2%) were female and 50% were aged between 19 and 35 years. Most users (57.9%) were male and the most widely represented age groups were middle-aged adults (40.8%), older children (26.3%) and adolescents (15.8%). The majority of users (67.1%) lived in the neighborhood where the school was located and 27.6% were aware of the role of the schoolyard as a climate shelter. As shown in Table 3, caretakers reported that schoolyards were mainly visited by families (adults with children of both genders aged under 12) and/or groups of friends consisting of adolescents and young adults (more boys than girls).

**TABLE 2:** Respondents’ characteristics of the spontaneous interviews. Climate Shelters in Schools, Barcelona, 2018–2022.

	Caretakers (N = 18)	Users (N = 76)
	n (%)	n (%)
Gender		
Female	13 (72.2)	32 (42.1)
Male	5 (27.8)	44 (57.9)
Age (years)		
Younger children (aged 0–5)	0 (0.0)	7 (9.2)
Older children (aged 6–11)	0 (0.0)	20 (26.3)
Adolescents (aged 12–18)	4 (22.2)	12 (15.8)
Young adults (aged 19–35)	9 (50.0)	1 (1.3)
Middle-aged adults (aged 36–55)	4 (22.2)	31 (40.8)
Older adults (aged >55)	1 (5.6)	5 (6.6)
Previous experience with the schoolyard before the intervention (yes)	6 (33.3)	30 (39.5)
Lives in the neighborhood (yes)		51 (67.1)
Belongs to the school community (yes)		17 (22.4)
Is aware of the schoolyard’s role as a climate shelter (yes)		21 (27.6)

**TABLE 3:** Schoolyard use and users’ and caretakers’ perceptions collected through brief spontaneous interviews. Climate Shelters in Schools, Barcelona, 2018–2022.

Category	Code	Quotations
User’s characteristics	• Adults with children (families) • Adolescents (groups of friends) • Higher number of boys	“The people who usually come here are families with children. Sometimes, teenagers also come and use the court to play football or basketball.” (22-year-old female caretaker) “More boys than girls come to the schoolyard.” (17-year-old male caretaker)
Thermal comfort and wellbeing	• Natural and artificial shade • Water for drinking and cooling off • Sense of thermal comfort and wellbeing • Improved air quality (vegetation) • Improved aesthetics (vegetation) • Comfort provided by seating areas • Significant contribution to the neighborhood (shade, water, vegetation, comfort)	“I feel good here, cool, and there are plants, and it’s pleasant.” (72-year-old female user) “We use [the water features] to drink and cool off when it’s very hot.” (6- and 9-year-old male users) “The perception of heat and wellbeing is quite good. It’s comfortable.” (17-year-old male caretaker) “It’s good because there is a lot of shade and, well, I like being in the shade where it’s not so hot and I do not sweat so much.” (8-year-old male user) “Vegetation is good because it offers a beautiful way to create shade.” (20-year-old female caretaker)
Activities and differences by age and gender	• Ball games and other sports played mainly by older children and adolescents • Large number of male participants in ball sports • Greater play diversification among younger children: playing with water, sandpit, slides, climbing • Sitting, chatting, and relaxing mainly by adults • Men play with their children more often	“Families with young children usually play in the children’s area, in the sandpit or sometimes with water, and those who are older usually play basketball or football in the court.” (22-year-old female caretaker) “I do not see any 20-year-old girls playing football or basketball here.” (48-year-old female caretaker) “Fathers and mothers sit in shaded areas or play with their children.” (27-year-old male caretaker)
Social behavior	• Meeting friends • Social play (children and adolescents) • Same-age and mixed-age playgroups • Verbal interaction with other users (parents) • Respect among users	“A friend of mine usually comes here and we play basketball together. And I also play with my father.” (9-year-old female user) “15- to 20-year-old boys usually come to play football (…). They let young children join in. It’s great. That way young children do not feel excluded.” (48-year-old female caretaker)
Additional benefits	• Friendly, car-free, and supervised • Clean • Closeness to home • Peacefulness, homely, not crowded • Better equipped than other public recreational areas	“I come here so my daughter can play and have space, be calm, and can meet other kids, and it’s not crowded … If I go to other parks in the city, there are usually more people. Here it’s more quiet and safer.” (48-year-old female user)
Areas for improvement	• Perception of heat • Insufficient shade, especially in the court • Lack of places to sit under the shade • Little, insufficiently grown, or poorly maintained vegetation • Dissatisfaction with fountain height, water jet, and number • Potential waste of water and safety concerns • Damaged equipment • Little diversity of playing areas • Insufficient provision of material and equipment • Underutilization and little dissemination	“They should look for a measure to really provide more shade in the sports court. It should be covered because it’s a large area of sunlight.” (53-year-old male user) “Some trees are missing water or…they look a little dry.” (13-year-old male user) “I like the fountain, but it would be better if you did not have to lower your head so much to drink and I would improve the water jet.” (10-year-old male users)

#### Thermal Comfort and Wellbeing

In general, users perceived the schoolyard as thermally comfortable and enjoyed the presence of shade areas provided by natural and artificial structures. Some respondents familiar with the pre-intervention schoolyards noted significant improvements in shade coverage after the transformations. According to one of the caretakers, the abundance of shade encouraged families to use the schoolyard. At some schools, caretakers observed an increase in new users visiting the schoolyards following the transformations. Users emphasized the importance of water features, especially during hot weather. These elements were considered effective for cooling off and staying hydrated and, according to caretakers, were mainly used by children and adolescents. Respondents noted that vegetation created a fresher, more pleasant environment, while creating more beautiful and naturalized spaces compared to pre-intervention schoolyards and improving air quality. Notably, respondents experienced a sense of wellbeing, and some expressed their satisfaction with the open schoolyards, finding them more comfortable than other neighborhood areas. This was particularly the case for the neighborhoods lacking green space, water, or shade. Adolescents and young adults stated they prefer the transformed over non-transformed schoolyard because of the shade and water structures (Table 3).

**FIGURE 1:**
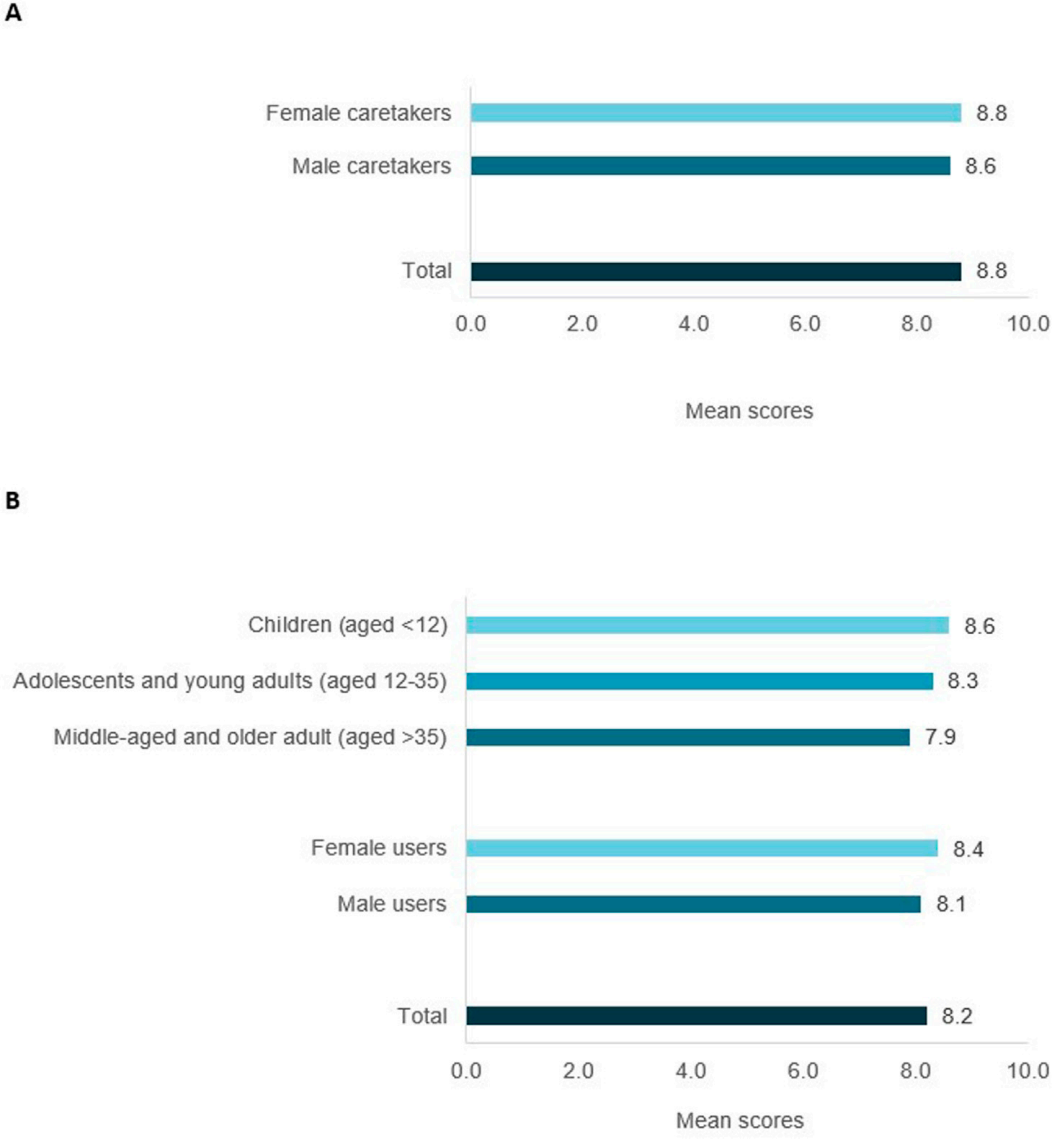
Participants’ mean scores regarding their perception of the suitability of schoolyards as climate shelter. **(A)** Caretakers’ scores by gender (N = 18); **(B)** users’ mean scores by age and gender (N = 76). Climate Shelters in Schools, Barcelona, 2018–2022.

Figure 1 shows respondents’ scores regarding their perception of the suitability of schoolyards as climate shelters. Caretakers’ mean score was 8.8 out of 10 while users’ score was 8.2.

#### Activities and Differences by Age and Gender

As shown in Table 3, caretakers noted that younger children tend to play a wider range of games than older children and adolescents, who usually engage in a single activity, mainly football or basketball. They also reported that users playing ball sports were usually boys. According to caretakers and some users, younger children usually engaged in activities such as playing in the sandpit, on the slides, or climbing. Another common activity, particularly among younger children, was playing with water. Adult users and caretakers stated that parents usually sat in shaded, seated areas, where they chatted with other users or relaxed.

#### Social Behavior

Respondents described the climate shelters as places for social gathering where they usually met friends and interacted with other families (Table 3). According to caretakers, while children, adolescents, and young adults played with their peers, parents usually interacted verbally with other users. Respondents also reported that adolescents occasionally let children of different ages join in their football games.

#### Additional Benefits

Users expressed their satisfaction with schoolyards being open to the public. Adults found them to be safe because, in contrast to other parks and playgrounds in the neighborhood, schoolyards were supervised by a responsible adult and were car-free. They also described the schoolyards as being peaceful, calm, clean, and uncrowded, making them a welcoming environment for their children. Another positive feature was their proximity to their homes. Finally, users emphasized that schoolyards were better equipped than other public recreational areas in the neighborhood due to the presence of sports courts, a diversity of areas, and comfortable facilities (Table 3).

#### Areas for Improvement

Despite giving positive feedback, respondents also suggested specific areas for improvement (Table 3). Both users and caretakers reported that some areas of the schoolyard were still hot, particularly the sports courts where there was little or no shade. In addition, adult users found that shaded seating areas were scarce in some schools. Respondents believed that vegetation was insufficiently grown or poorly maintained and suggested increasing the amount of greenery. Although water features were among the most widely used and appreciated interventions, concerns were raised about the fountains being too high for younger children and the water jets being too strong. Users also suggested increasing the number of these elements. Some of them expressed additional concerns about potential water waste and the puddles that are sometimes formed.

Some adults requested more varied play spaces and additional materials. Lastly, some respondents stressed that information on the program should be more widely disseminated to increase utilization of these schoolyards (Table 3).

## Discussion

Transforming schoolyards into publicly accessible climate shelters through the provision of vegetation, water, and shade provides a unique opportunity for urban adaptation to climate change. This study provides evidence of the benefits of this initiative, demonstrating the potential of schoolyards to provide urban local communities with areas for cooling and relaxation while offering infrastructure that encourages outdoor recreation and social interaction in safe and welcoming spaces.

In this study, both the observational data and caretakers’ reports indicated that the schoolyards were mainly used by families with children and groups of adolescents. This finding may be influenced by the schoolyards’ designs, which incorporate features and amenities tailored to younger users. While older adults can also benefit from this type of initiative, a small number were observed visiting the schoolyards. Possible reasons for their low numbers are that this age group may not be fully informed about the availability of these spaces as climate shelters [21] or may prefer visiting outdoor green spaces without the presence of young people [22] or outside of afternoon hours [23].

In line with the findings of previous studies on the use of outdoor public spaces [24–26], our results showed that boys used the schoolyard more than girls outside school hours, especially among adolescents and young adults. In this study, we found that the presence of sports courts may attract adolescent boys to the schoolyard since they reported that these facilities were one of their favorite features. However, these facilities might appeal less to girls [26–28], which may explain why they tend to visit these spaces less frequently outside school hours. These results highlight the need to encourage the utilization of open schoolyards among adolescent girls. Prior literature suggests programming can enhance parks’ inclusivity by increasing accessibility, flexibility, relatability, and sociability [29]. Strategies to increase schoolyards’ usage among adolescent girls should focus on implementing targeted community programs that increase access to organized activities or planned events that offer a wide range of recreational activities tailored to their interests. An additional initiative could be actively involving adolescent girls in the design process to understand their needs and preferences.

In the present study, users and caretakers showed high levels of satisfaction with the schoolyards as climate shelters. They appreciated the shade, water, and vegetation, which reduced perceived thermal discomfort and made the areas more comfortable than other neighborhood spots and non-transformed schoolyards. Despite limited pre-intervention data, our finding suggests that a subset of respondents observed significant improvements in post-intervention schoolyards when compared to their previous state. The provision of greater shade coverage, water features, and vegetation appears to have created more inviting environments. Observational data showed that fountains and evaporative misters for cooling purposes were frequently used by young users, as were shaded and seating areas for relaxation by adults. These results add to a growing body of evidence demonstrating the potential of greenery, water features, and shade to reduce heat stress and improve urban thermal comfort [11, 12, 14, 17, 30]. Overall, our findings support the idea that using multiple cooling measures in urban schoolyards is a promising strategy for creating outdoor areas where the local community can comfortably enjoy spending time.

Supported by previous work [18, 19], we found that climate-adapted schoolyards also provided opportunities for outdoor recreation outside school hours, especially for children and adolescents. In line with prior research [31–33], our findings suggest that the presence of sports courts are important for young people’s engagement and physical activity in urban green spaces. Also in line with other studies [25, 26, 28, 31], we found a gender gap in the use of such areas, with use being higher among boys. Our study also revealed that younger children tend to engage in a wide diversity of activities and use different spaces (e.g., resting areas, playground, court) during their visits. Notably, water fountains were one of the favorite features for younger children to play with, indicating the benefits of such features not only for cooling but also for children’s enjoyment.

Recent work has suggested the potential of green schoolyards to become areas for social engagement [18, 19]. Our results show that seating and shaded areas encouraged verbal interaction among schoolyard users, particularly parents but also adolescents and young adults. These findings are in agreement with a previous study reporting that adults often socialized on benches in Mediterranean urban playgrounds [27]. Another study reported that seating and shaded areas, as well as sports courts, encouraged socialization among adolescents in parks [28]. In this study, we found sports courts and playground areas in the schoolyards encouraged social interaction by fostering social play among groups of young people of both similar and different ages. Social interaction during outdoor play and mixed-age play can positively impact young people’s socio-emotional development and social cohesion, potentially extending to their parents [16, 34]. Thus, our findings highlight the value of designing schoolyards as community spaces with a diversity of areas that may encourage various types of socialization among different population groups, providing opportunities for community bonding.

Characteristics such as perceived safety and cleanliness have been linked to greater use of community parks [26, 35, 36]. Thus, a positive finding of our study is that parents perceived the schoolyards as safe (car-free and supervised), peaceful, and clean. Indeed, they cited these factors as reasons for choosing the transformed schoolyards over other community areas. Another factor attracting users to the schoolyards was their close proximity to home, supporting the need to increase the number of urban climate-adapted recreational spaces near residents’ homes. This finding is consistent with previous research reporting a link between residential proximity and greater park use [24].

The results of this study indicate that potential improvements of the intervention include increasing shade coverage in sports courts which could enhance schoolyard users’ thermal comfort, as evidenced by both respondents’ and observers’ perceptions of heat, particularly in these areas. In addition, greater efforts are needed to maintain vegetation in good condition. Some respondents also raised concerns about potential water waste, which is particularly significant in cities facing water shortages, such as Barcelona. Improving the efficiency of water interventions and promoting responsible water use among residents could help provide cooling opportunities while minimizing water waste. Respondents’ suggestions for improvement include increasing the amount of vegetation, as well as the number of fountains and shaded seating areas. Tackling these issues may improve users’ enjoyment and comfort in schoolyards.

Our results also indicated potential underutilization of the schoolyards, with the number of users observed ranging from 0 to 24. Limited use of schoolyards outside school hours has already been reported in the U.S. [37]. Our findings could be partly explained by low awareness of the program among city residents. This explanation is supported by previous research suggesting that Barcelona residents have little awareness of the municipal climate network [21], as well as our finding that most interviewees reported they were unaware of the schoolyard’s role as a climate shelter. Therefore, greater dissemination of the program could increase the use of schoolyards as climate shelters. To achieve this, the local government, educational institutions, and community organizations must collaborate. Strategies may include raising awareness through community events, targeted communication campaigns, and actively involving the local community in decision-making processes. Another reason that may contribute to the low number of visits to schoolyards could be that the transformed schoolyards do not currently seem to accommodate the needs of all population groups, as evidenced by the small number of older people in our study. In this regard, an important consideration is that while these spaces are open as a community climate shelter during off-school hours, schoolyards are primarily designed and adapted to accommodate children’s needs.

This study is limited by the absence of a comparison group and pre-intervention data. However, some data is available from users who were familiar with the schoolyard before the interventions and perceived changes after the transformations. No comparable schools were opened to the community during data collection (summer 2022). In addition, data collection was restricted to 2 visits per school, which could limit the generalizability of the results. To minimize potential biases, we ensured consistent timing (afternoon visits), used standardized protocols, avoided certain weather conditions (e.g., rainy days), and conducted weekend and weekday observations to account for potential fluctuations in patterns of use. Furthermore, we enriched the observational data with interviews from informants such as caretakers, who provided a broader perspective on the usual schoolyard dynamics. Finally, our qualitative approach offered valuable insights into participants’ experiences with the intervention and allowed us to understand the potential effects of specific elements (i.e., vegetation, water, shade, seats) and how the intervention could be improved.

This study adds to existing knowledge by evaluating an innovative project (“Climate Shelters in Schools”) to demonstrate how combining water, vegetation, and shade to schoolyards and opening them to the public can create climate-resilient settings that enhance the wellbeing of the local community. The study offers valuable insights for policymakers and urban planners to optimize the design and implementation of these types of climate adaptation strategies, especially in cities facing challenges similar to those in Barcelona. This study also complements previous research assessing the impact of the same intervention at the school-level [20], offering a broader perspective on its benefits.

Another strength of the study is the use of different methods to gain deeper understanding of schoolyard use and perceptions of the intervention. We included the perceptions of people of different ages and genders and incorporated the viewpoint of community members as well as schoolyard caretakers.

### Conclusion

The schoolyards transformed as part of the “Climate Shelters in Schools” project can positively impact the local community, creating safe and convenient spaces where residents can feel comfortable and protected from the heat. The diversity of features and areas in the schoolyards can encourage recreation, exercise, and social interaction, particularly among adolescents, children, and their parents. While the benefits seem clear, this study also underlines the need to maintain greenery, improve water sustainability, and provide greater shade coverage in future implementations. The successful implementation of these projects requires effective dissemination strategies to maximize their impact. Overall, we provide policymakers with evidence to guide the adaptation of schoolyards into climate shelters, which can benefit the local community by providing comfortable recreational environments.
