# Supplementary material for: Community Use and Perceptions of Climate Shelters in Schoolyards in Barcelona
Source: Int J Public Health. 2025 Feb 7;70:1608083. doi: 10.3389/ijph.2025.1608083 (PMC11842932; doi:10.3389/ijph.2025.1608083)
Supplement: Supplementary file 1 [file DataSheet1.pdf]

Supplementary data

Figure S1. Photographs captured during the observation sessions showing examples of the interventions implemented in the schoolyards (water features, vegetation, shade structures, and seating areas). Climate Shelters in Schools, Barcelona, 2018-2022.

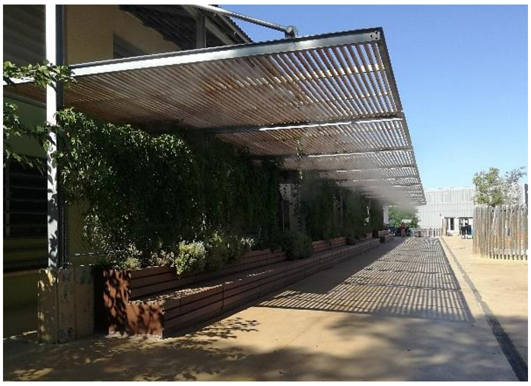

Image 1. Pergola with an evaporative mister combined with vegetation and

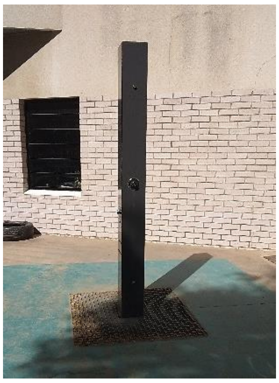

Image 2. Fountain for playing and cooling

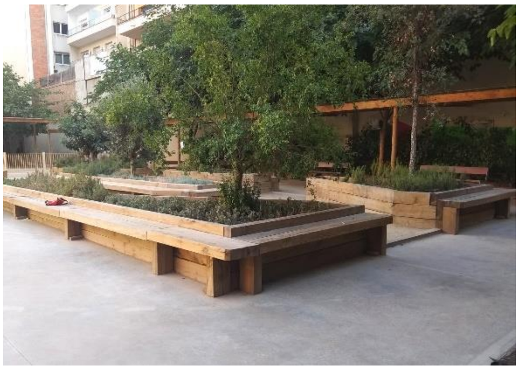

Image 3. Trees and planters with Mediterranean species combined with

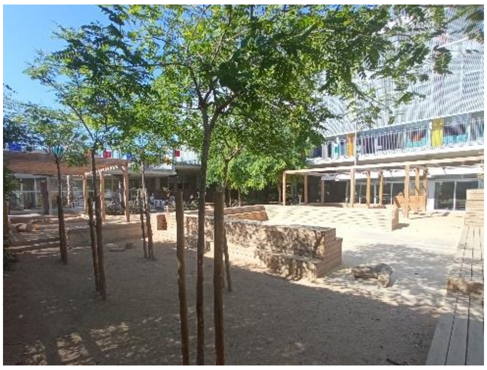

Image 4. Trees, pergolas, and wooden seating areas with sandy ground

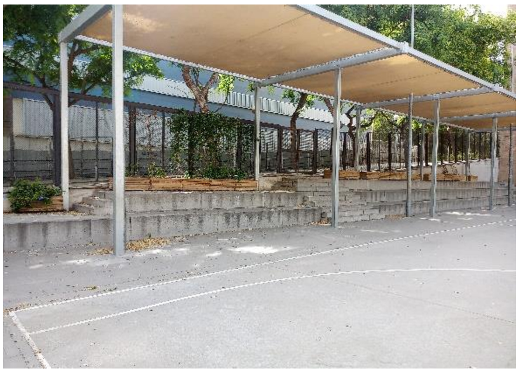

Image 5. Canopy located in a seating area next to the sport court

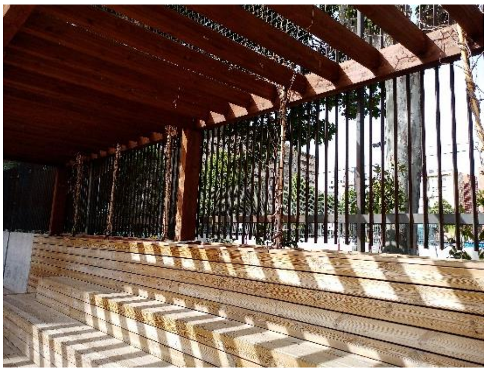

Image 6. Pergola combined with wooden tiered seating
